# Alcohol use during the COVID-19 pandemic: gender, parenthood, intimate partner violence, and stress

**DOI:** 10.3934/publichealth.2023027

**Published:** 2023-05-09

**Authors:** Kassidy C Colton, Stephanie A Godleski, Joseph S Baschnagel, Rebecca J Houston, Shine M DeHarder

**Affiliations:** Health and Addictions Research Center, Department of Psychology, Rochester Institute of Technology, Rochester, NY 14623, USA

**Keywords:** COVID-19, pandemic, parents, intimate partner violence, alcohol use, stress

## Abstract

Some preliminary work during the COVID-19 pandemic indicates that adult alcohol use increased, particularly for parents. This cross-sectional study examined the quantity and frequency of adults' alcohol use during the early stages of the pandemic. Additionally, the influences of gender, parenthood, COVID-19-related stressors and intimate partner violence (IPV) on alcohol consumption were examined. The sample consisted of 298 adults (98 parents) from across the United States who completed self-report surveys through Qualtrics at the beginning of the pandemic in May 2020. In the present study, all men reported higher levels of drinking compared to all women. Although stress levels did not impact alcohol consumption, findings indicate that increased IPV experiences were associated with higher levels of heavy drinking during the pandemic. Results also suggested that having children in the home particularly impacted drinking levels during the pandemic, above and beyond the influence of gender, IPV, and stress levels. These findings suggest that parenthood may have had a cascading influence on drinking experiences during the COVID-19 pandemic. Implications and recommendations for further research are discussed.

## 1. Introduction

In March 2020, the World Health Organization (WHO) declared a national emergency regarding the Coronavirus (COVID-19) pandemic—a widespread, global health concern that brought forth unprecedented challenges. Collectively, the impacts and persistent demands at the beginning of the COVID-19 pandemic significantly affected the psychosocial functioning and well-being of many individuals and families [1]–[5]. Notably, individual drinking rates significantly increased during the mandated COVID-19 quarantine [6]–[10], potentially as a means to cope with the accompanying social isolation [11]. Heavy alcohol use (i.e., 4 or more drinks on any day, or more than 7 drinks per week, for women; 5 or more drinks on any day, or more than 14 drinks per week, for men [12]) is associated with financial [13], psychological [14], and health consequences [15]. Considering its health significance, the purpose of the current study was to examine the contextual risk factors associated with heavier levels of alcohol consumption during the COVID-19 pandemic, including parenthood, gender, stress, and intimate partner violence (IPV; violence and aggression between intimate partners). An additional goal of the present study was to examine whether the relationship between parenthood and alcohol use during the early stages of the pandemic depends on one's gender identity.

### 1.1. Impact of stress

Stress is often implicated in the onset and maintenance of alcohol problems [16],[17]. Experiencing stress (e.g., financial, psychological, etc.) may increase alcohol consumption due to alcohol's short-term tension-reducing properties [18],[19]. Indeed, the early months of COVID-19 posed numerous stressors including economic difficulties [20] and uncertainty about the illness [21], both of which likely contributed to increased alcohol consumption. In fact, Grossman and colleagues [6] report that increased stress was the one of the biggest reason adults drank during the early months of COVID-19. Therefore, it was expected that financial and illness-related stress during the pandemic would increase alcohol use among adults in the present study.

### 1.2. Impact of IPV

Several forms of IPV perpetration and victimization (i.e., physical, verbal, and sexual) have been positively associated with alcohol consumption [22]–[25]. Although the link between perpetration and alcohol use has garnered more empirical evidence than victimization [26],[27], the self-medication hypothesis suggests that alcohol is often used as a coping strategy to distract victims of violence from associated psychological distress [28]–[30]. Weinsheimer et al. [31] found that nearly two-thirds of couples with past-year IPV experiences (i.e., both perpetration and victimization) also reported heavy episodic drinking. Furthermore, a meta-analysis revealed that physical IPV was most closely associated with alcohol misuse compared to sexual and psychological IPV [32]. Conflict and violence among couples and families has also been shown to be exacerbated during major crises and times of economic hardship [33]. This has remained true regarding COVID-19, such that in some regions of the United States (i.e., Northeast and West), domestic violence-related police calls [34] and incidents [35],[36] significantly increased at the beginning of the pandemic. Moreover, physical IPV was estimated to be 1.8 times greater during the stay-at-home orders when more time was spent with a partner but isolated from others outside of the household [37]. Thus, it was expected that physical IPV experiences during the beginning of COVID-19 would be associated with higher levels of drinking.

### 1.3. Gender differences

Several lines of evidence suggest that there are gender differences regarding experiences of alcohol use. Men typically report consuming higher amounts of alcohol compared to women [38]–[40], and some evidence suggests that being a man was a risk factor for increased heavy episodic drinking during the first few months of the pandemic [7],[41]. Given existing gender differences in alcohol use, as well as the potential impact of the pandemic on men's heavy episodic drinking, it was expected that men would report drinking higher quantities and more frequently than women during the pandemic stay-at-home orders.

### 1.4. Impact of parenthood

It is crucial to understand the influence of parenthood on alcohol use during the early stages of the COVID-19 pandemic, as alcohol consumption by parents often has a cascading influence on families through parenting practices (e.g., [42]) and child behavior problems (e.g., [43]). For instance, parental alcohol use is positively associated with harsh caregiving behaviors [44], and such parenting practices increase children's risk of emotional and behavior problems [45]. With regards to COVID-19, pandemic-related restrictions increased childcare responsibilities for many parents, potentially exacerbating the impact of the COVID-19 pandemic on parents' alcohol use [4],[7],[8]. There is some research both prior to [46]–[49] and during [50] the pandemic indicating that parents are more likely to consume alcohol than adults without children. With the COVID-19 pandemic imposing stay-at-home restrictions and thus additional childcare responsibilities for parents, it is likely that their drinking increased at higher rates than adults without children. Therefore, it was expected that adults with children would consume more alcohol during the pandemic than adults without children.

### 1.5. Differences between mothers and fathers

There are also documented gender differences between mothers and fathers, with fathers consuming larger quantities of alcohol and drinking more frequently than mothers [46],[51]. In addition, preliminary COVID-19 research found that fathers reported significantly higher alcohol consumption than did mothers [52],[53], suggesting that the pandemic stay-at-home orders may have placed fathers at greater risk for heavier alcohol use than mothers. Although identifying as a man alone is associated with heavier alcohol use [39], the additional responsibilitiy of being a parent may increase the likelihood of problematic alcohol consumption for fathers during the pandemic [52]–[54]. Thus, it was expected that fathers in the present study would report consuming higher quantities of alcohol and drinking more frequently than mothers during the pandemic.

### 1.6. Current study

Based on the above review, the primary goal of the current study was to examine the incremental influence of stress, IPV, gender, and parenthood on alcohol use during the COVID-19 pandemic (e.g., [55]). Notably, many researchers have highlighted the need for this research [56]–[58], and this study intends to bridge that gap in the extant literature. It was expected that higher levels of stress, increased IPV experiences, identifying as a man, and having children in the home at the beginning of the COVID-19 pandemic would all be associated with higher levels of alcohol use.

Additionally, there is a great deal of evidence suggesting that the relation between parenthood and alcohol use might depend on one's gender. Thus, the present study also sought to examine whether fathers or mothers were at the greatest risk for alcohol use at the beginning of the COVID-19 pandemic. It was further hypothesized that gender would moderate the association between parenthood and alcohol use such that fathers would report higher levels of alcohol use than mothers.

## 2. Methods

### 2.1. Participants

The participants were 298 adults from across the United States. Demographic characteristics of all participants can be found in Table 1. The sample was recruited as part of a larger study examining the impact of COVID-19 on health and behavior in May 2020. Individuals were deemed eligible to participate if they were at least 18 years of age and lived in the United States. Participants were specifically recruited through a Qualtrics panel, which compensates participants with points that they can redeem for rewards. See Miller et al. [59] and Belliveau et al. [60] for review of Qualtrics panel procedures. Participants in the present study received approximately $4.40 in reward points as compensation.

### 2.2. Measures

#### 2.2.1. Alcohol consumption

To assess alcohol consumption during the COVID-19 pandemic, the quantity-frequency index of alcohol use was used [61]. This is a common approach to estimate the average alcohol consumption for an individual [62]–[64]. To derive the quantity-frequency index, two items assessed participants' quantity (“On average, how many days per week did you drink alcohol?”) and frequency (e.g., “On a typical drinking day, how many drinks did you have?”) of alcohol use during the pandemic. Both of the items were significantly correlated (*r* = 0.66, *p* < 0.001) and were multiplied by each other to create an index of alcohol use. The quantity-frequency index of alcohol use during the pandemic (i.e., COVID QFI) was used in the primary analyses.

#### 2.2.2. Intimate partner violence

Experiences of IPV are frequently bidirectional [65]. Because the goal of the present study was to examine the overall experience of physical IPV within relationships, dyadic experiences of both victimization and perpetration were considered following similar approaches by previous studies [66]–[68]. As such, experiences of physical IPV were measured through a rapid IPV screening tool developed and proposed by Crane and colleagues [69]. This IPV screening measure was adapted from the Revised Conflict Tactics Scale Short Form [70] and contains eight total items on both perpetration and victimization of physical IPV. Four items assess perpetration (e.g., “I slapped my partner”) and four items assess victimization (e.g., “My partner slapped me”) of physical IPV within the past 30 days in the relationship. All items are dichotomously rated (1 = *yes*, 0 = *no*) regarding whether the behavior was experienced or not. The perpetration and victimization scales were highly correlated (*r* = 0.74, *p* < 0.001) and the eight items were summed to obtain total physical IPV experience scores (range 0 to 8). Higher scores indicate more experiences of physical violence within the relationship in the past 30 days (Cronbach's *α* = 0.88).

#### 2.2.3. Current stress

Participants completed the Perceived Stress Scale-10 [71], which comprises 10 items that evaluate participants' stressful feelings over the last month (e.g., “In the last month, how often have you felt nervous and ‘stressed’?”). All items are rated on a 5-point scale (0 = *Never*, 4 = *Very Often*). Total summed scores across all 10 items were used (range 0 to 50), with higher scores indicating more stressful experiences in the previous month (Cronbach's *α* = 0.85).

#### 2.2.4. COVID-related stress

In the present study, COVID-related stress includes both financial and illness stressors. To evaluate COVID-related financial stressors, participants reported on two questions regarding their change in work status during the pandemic (0 = *No change since pandemic*, 1 = *Unemployed/laid off from work due to pandemic*) and perceptions of financial difficulty related to COVID (0 = *Not at all difficult*, 1 = *Somewhat to Extremely difficult*). These two items were summed to create a COVID financial stress variable (range 0 to 2), with higher scores reflecting more financial stress due to the pandemic.

Additionally, five items were used to assess COVID-19 illness stressors. Participants reported separately on whether they or anyone in their household is considered at high risk (0 = *No*, 1 = *Yes*), as well as whether themselves, a friend, or a family member had received a COVID-19 diagnosis (0 = *No*, 1 = *Yes*). Answers on all five items were summed to create an overall COVID illness stressor variable (range 0 to 5), with higher scores reflecting more illness-related stressors.

#### 2.2.5. Parenthood

Participants were asked whether or not they had children. Those that reported having children were asked the ages of their children and whether their children lived with them. For the purposes of the study, participants who reported currently having children under the age of 18 years old living with them and under their care were considered to have children in the home.

### 2.3. Procedure

The study materials and procedures were approved by the Rochester Institute of Technology Institutional Review Board. Participants were recruited via Qualtrics panels through which they received direct compensation. Informed consent was obtained online from all participants through the Qualtrics software prior to completing the survey material on their own personal devices. Confidentiality and anonymity of all collected data were assured. Participant data were excluded if responses were considered poor quality based on failed attention checks, incoherent responses on open ended questions, or if particpants completed the survey in less than a third of the median survey duration. Of 389 participants who began the survey, 91 were excluded, leaving a final sample of 298.

### 2.4. Statistical approach

All data were subject to extensive cleaning prior to analyses [72], and all analyses were completed using SPSS Version 28.0 [73]. Descriptive statistics were calculated in order to assess for bivariate correlations and means of all study variables. Then, independent samples *t*-tests were used to compare parents (i.e., having children under 18 at home) to those without children under their care on stress levels, IPV experiences, and alcohol consumption. Additionally, we examined gender differences in alcohol use. A hierarchical regression was conducted to test the incremental influence of parenthood and gender, as well as their potential interaction, on alcohol use during the pandemic, covarying for IPV and stress levels. In the first step of the model predicting quantity and frequency of alcohol use, current and COVID-19 related stress levels were entered. Next, IPV and gender were entered in the second and third steps, respectively. Then, parent status was entered in the fourth step of the model, followed by the interaction term between gender and parenthood in the final step.

## 3. Results

### 3.1. Preliminary analyses

Bivariate correlations and descriptive statistics of all study variables are presented in Table 2. The reported number of alcoholic drinks consumed per day for the particpants in the present sample ranged from zero to 15, and reported drinking days per week ranged from zero to seven. On average, participants reported drinking 1.77 drinks a day and drinking 2.08 days a week during the COVID-19 pandemic. Number of alcoholic drinks per day during the pandemic was associated with higher stress levels (*r* = 0.21, *p* < 0.01) and IPV experiences (*r* = 0.31, *p* < 0.001). In addition, more frequent drinking was associated with higher rates of IPV (*r* = 0.19, *p* < 0.05).

Mean differences by parent status, as well as differences for parents by gender, among all study variables are presented in Tables 3 and 4. Men in the sample reported significantly higher levels of daily and weekly drinking in comparison to women, *t*(222) = 3.24, *p* < 0.001 (*M_men_* = 11.07, *M_women_* = 5.44). Parents reported higher levels of IPV, *t*(113) = −2.58, *p* = 0.011, COVID-related financial stress, *t*(170) = −4.29, *p* < 0.001, current overall stress levels, *t*(168) = −5.57, *p* < 0.001, and both daily, *t*(136) = −6.03, *p* < 0.001, and weekly alcohol consumption, *t*(136) = −5.13, *p* < 0.001, than adults without children under their care. However, parents reported significantly less COVID-19 illness-related stress than those without children under their care, *t*(167) = 3.14, *p* = 0.002. Gender difference tests for only the parents in the sample revealed that fathers reported more physical IPV perpetration and victimization than mothers, *t*(81) = 2.45, *p* = 0.016. However, mothers and fathers did not differ on reports of drinking, *t*(75) = 1.00, *p* = 0.320, current stress, *t*(94) = 0.12, *p* = 0.669, COVID-19-related financial stress, *t*(96) = −0.45, *p* = 0.488, or illness stress, *t*(93) = 0.67, *p* = 0.350.

**Table 1.** Participants' sociodemographic characteristics.

Characteristics	Frequency	%	*M* (*SD*)
Age (years)	-	-	49.18 (17.42)
18–24	26	8.72	-
25–34	48	16.11	-
35–44	59	19.80	-
45–54	36	12.08	-
55–64	46	15.44	-
65+	83	27.85	-
Parent Status			
Children under 18 at home	98	32.89	-
Children over 18 at home	38	12.75	-
No children	162	54.36	-
Gender			
Man	99	33.22	-
Woman	199	66.78	-
Gender by Parent Status			
Father	44	44.90	-
Mother	54	55.10	-
Race			
White	251	84.23	-
Black or African American	24	8.05	-
Asian	18	6.04	-
American Indian or Alaskan Native	5	1.68	-
Household Income from prior 12 months			
$0	44	14.77	-
$2500–$4499	15	5.03	-
$5000–$9999	11	3.69	-
$10,000–$14,999	15	5.03	-
$15,000–$22,499	26	8.73	-
$22,500–$29,999	24	8.05	-
$30,000–$39,999	33	11.07	-
$40,000–$49,999	35	11.74	-
$50,000 or more	95	31.89	-
U.S. region			
Northeast	73	24.49	-
Midwest	67	22.48	-
South	106	35.58	-
West	52	17.45	-

**Table 2.** Bivariate associations and descriptive statistics among study variables.

	1.	2.	3.	4.	5.	6.
1. IPV	-					
2. Stress	0.24**	-				
3. Alcohol Use – Number of Drinks/Day	0.31***	0.21**	-			
4. Alcohol Use – Number of Drinking Days/Week	0.19*	0.04	0.66***	-		
5. COVID Financial Stress	0.11	0.36***	0.17	0.07	-	
6. COVID Illness Stress	0.35***	0.02	0.03	0.02	0.10	-
Mean	0.53	16.55	1.77	2.08	0.76	1.13
*SD*	1.46	7.72	2.28	2.36	0.62	1.09

*Note: *p < 0.05; **p < 0.01; ***p < 0.001.

**Table 3.** Mean differences by parent status.

	Children (*n* = 98)	No Children/Adult Children (*n* = 74)
IPV	0.95 (1.93)*	0.06 (0.35)*
Stress	19.18 (7.13)*	12.81 (7.72)*
Alcohol Use – Number of Drinks/Day	2.87 (2.69)*	0.67 (1.06)*
Alcohol Use – Number of Drinking Days/Week	2.90 (2.29)*	1.03 (1.88)*
COVID Financial Stress	0.92 (0.64)*	0.53 (0.53)*
COVID Illness Stress	0.91 (1.26)*	1.46 (0.97)*

*Note: Standard deviations are provided in parentheses; *Indicates significant differences between pair, *p* < 0.05.

**Table 4.** Mean differences for parents by gender.

	Father (*n* = 44)	Mother (*n* = 54)
IPV	1.49 (2.44)*	0.48 (1.17)*
Stress	19.27 (7.25)	19.10 (7.10)
Alcohol Use – Number of Drinks/Day	3.32 (3.04)	2.45 (2.28)
Alcohol Use – Number of Drinking Days/Week	3.14 (2.16)	2.68 (2.41)
COVID Financial Stress	0.89 (0.65)	0.94 (0.63)
COVID Illness Stress	1.00 (1.43)	0.83 (1.10)

*Note: Standard deviations are provided in parentheses; *Indicates significant differences between pair, *p* < 0.05.

### 3.2. Primary analyses: Predictors of alcohol use

The results of the hierarchical regression analysis are presented in Table 5. In the first step of the regression model, the set of stress variables did not explain a significant amount of variance in participants' quantity and frequency of alcohol use, [*R^2^* = 0.03, *F*(3,94) = 1.01, *p* = 0.394]. In the next step, levels of IPV reported within dyads explained a significant amount of variance in alcohol consumption, [Δ*R^2^* = 0.08, Δ*F*(1,93) = 18.48, *p* = 0.004], above and beyond stress. Higher levels of reported IPV perpetration and victimization were associated with increased alcohol use (*β* = 0.33). However, gender did not explain a significant amount of variance above and beyond that of IPV and participants' stress, [Δ*R^2^* = 0.02, Δ*F*(1,92) = 2.10, *p* = 0.151]. In the fourth step, having children under 18 living in the home further explained a significant amount of variance in alcohol use above and beyond stress, IPV, and gender, [Δ*R^2^* = 0.09, Δ*F*(1,91) = 10.14, *p* = 0.002]. Having a child in the home uniquely predicted alcohol use such that having children under 18 at home (*β* = 0.37) was significantly associated with higher levels of alcohol consumption during the pandemic. Finally, the interaction between gender and parenthood in the final step of the model did not explain a unique amount of variance in alcohol use, [Δ*R^2^* = 0.01, Δ*F*(1,90) = 1.04, *p* = 0.310].

**Table 5.** Summary of a hierarchical regression model predicting alcohol use during the pandemic.

Step	Variable	*β*	*R^2^*	Δ*R^2^*
1	Current Stress	0.11	0.03	0.03
	COVID Financial Stress	−0.01		
	COVID Illness Stress	0.13		
2	IPV	0.33*	0.11	0.08
3	Gender	−0.15	0.13	0.02
4	Parenthood	0.37*	0.22	0.09
5	Gender X Parenthood	0.27	0.23	0.01

*Note: Gender was coded as 0 = Man, 1 = Woman; Parenthood was coded as 0 = No children at home or adult children, 1 = children under 18 living at home; *p < 0.001.

## 4. Discussion

With the COVID-19 pandemic imposing abrupt changes to individuals' daily lives, the goal of this study was to understand how overall and pandemic-related stress, IPV, gender, and parenthood impacted alcohol consumption during the early stages of COVID-19. An additional goal of this study was to examine whether the association between parenthood and alcohol use was stronger for fathers compared to mothers. Importantly, having children in the home during the pandemic predicted greater levels of alcohol use above and beyond gender, IPV experiences, and stress. As anticipated and consistent with past research [7], men reported more drinks per day and drinking days per week compared to women. Parents in this sample also reported more frequent and higher levels of drinking than adults without children. However, the interaction between gender and parenthood was not significant in predicting alcohol use during the pandemic. Findings are discussed below in the context of existing literature.

In the present study, all men reported higher levels of drinking compared to all women, suggesting that identifying as a man may have been a risk factor for heavier alcohol use at the beginning of the pandemic. This is consistent with our hypotheses and prior research conducted before [39] and during the pandemic [74]. It is possible that men are at an increased risk for heavier alcohol use because they tend to have less adaptive coping mechanisms compared to women [75],[76]. Furthermore, men tend to report higher levels of drinking to cope with stress compared to women [11]. However, gender was not a significant predictor of alcohol use within the context of other potential social and situational influences (i.e., stress, IPV, and parenthood), which is inconsistent with our hypotheses. A possible explanation for this might be that during the COVID-19 pandemic, other factors (i.e., IPV and parenthood) were more significant in accounting for alcohol use. In fact, and not surprisingly, we also found that experiencing IPV was a significant risk factor for drinking at higher levels during the pandemic. This finding is consistent with theory and previous findings [77],[78]. It may be that men were more likely to experience IPV and thus, drank at higher levels than women as a means to cope [79],[80]. Collectively, results suggest that experiencing IPV may have a greater influence on frequency and quantity of alcohol consumption.

Perhaps the most notable finding of the present study is that parenthood was a significant risk factor for heavy alcohol use during the pandemic, above and beyond the influence of gender, stress, and IPV. Compared to adults without children, parents consumed significantly more alcohol in the early stages of the pandemic. This is consistent with both hypotheses and initial research from the pandemic [50],[81]. Indeed, Schmits and Glowacz [74] demonstrated that having children was a significant indicator of increased alcohol use during COVID-19 in a Belgian sample. Although there is some work suggesting that parents drink less than adults without children (e.g., [82],[83]), Bowden et al. [46] found that parents are more likely to drink at home. This may have been the case during the COVID-19 pandemic [84]. Parczewska [85] further suggests that parents may have lacked necessary coping skills or support when taking on multiple roles (e.g., teacher, caregiver, etc.) at the beginning of the pandemic. This may have further increased alcohol consumption for parents. Interestingly, there were no significant differences between the mothers and fathers in the sample on alcohol use. The impact of parenting children during the early part of the pandemic may have influenced alcohol consumption similarly for both mothers and fathers. This was further supported by the lack of a significant interaction between gender and parenthood status in predicting alcohol use. Although gender differences on alcohol use exist in the present study and prior work, these differences may dissipate when one's parent status is considered. Accordingly, being a parent may have been a risk factor for drinking during the pandemic regardless of gender [86]. In fact, the only difference between mothers and fathers in the present study was that fathers reported more experiences of IPV in their relationship compared to mothers.

It was surprising that neither of the COVID-related stress variables or overall stress were associated with alcohol consumption, which conflicts with our expectations and prior work during the pandemic (e.g., [87]). In a Canadian sample, Thompson et al. [41] found that increased stress and emotional distress due to the pandemic was associated with drinking more frequently, but only among the men in their sample. With the men in our study reporting higher mean levels of alcohol use than women, it is likely that the relationship between stress levels and alcohol use depends on one's gender. In addition, Adams et al. [88] found that parents accounted their stress levels to COVID-related stressors, similar to our finding that parents in the present study reported higher COVID-related stress than adults without children. Although their sample contained parents only, it could be that the relationship between stress and alcohol use during the pandemic also depends on one's parent status. For example, Portugese parents experienced significantly higher levels of burnout and stress compared to adults without children during the pandemic [89]. Furthermore, Tucker et al. [90] found that only social stress (e.g., stress from being lonely) at the beginning of the pandemic in the U.S predicted increased daily alcohol consumption longitudinally. Therefore, only specific domains of stress experienced during COVID-19 may be associated with problematic alcohol consumption (i.e., social stress) and not others (i.e., general stress, financial stress, stress about the virus). Taken together, the association between stress and alcohol use during the pandemic may be complex and dependent on many other factors (i.e., gender, parenthood, type of stress).

As always, this study is not without limitations. The cross-sectional nature of the data collection meant pre-pandemic measures were unavailable for some variables, which meant we were unable to measure how individuals' alcohol consumption may have changed from before the pandemic. Additionally, the data was collected at the beginning of the pandemic in the United States during a time when much was unknown about risks of contracting the COVID-19 virus. Due to this, we cannot assess how the relationships between these variables may have changed with varying COVID-19-related restrictions and safety measures. In addition, the current study did not consider the influence of the number of children at home or child age. Stress levels (e.g., [91]) and alcohol use (e.g., [46]) may differ for parents with multiple children and for parents of younger children who require more supervision and help with schoolwork. Subsequent research should focus on these family characteristics to explore a more nuanced understanding of these associations. Furthermore, parents may be at greater risk of experiencing stress and burnout from the pandemic given the unique disruptions to their daily lives (i.e., unexpected childcare responsibilities; [88],[92]). Thus, future work should examine the mechanisms through which parents are at higher risk to misuse alcohol during major life disruptions, such as through the influence of increased stress. These findings also suggest it may be beneficial to examine the role of coping and emotion regulation strategies as they relate to gender, IPV, alcohol consumption, and stress during the pandemic [93]. Finally, although results did not differ if considered separately in the present study, future work could consider distinguishing between predictors of perpetration and victimization when investigating the influence of COVID-19 on experiences of other types of IPV in partner relationships (e.g., [94]).

## 5. Conclusions

Altogether, this study highlights various factors that may have contributed to alcohol use during the pandemic. To our knowledge, no other study simultaneously considers stress, IPV, gender, and parenthood in alcohol consumption during the pandemic. Results found that men reported significantly higher mean levels of drinking in comparison to women. Findings from the present study also suggest that experiencing physical IPV and being a parent at the beginning of COVID-19 was associated with higher alcohol use. While this study focused on the effects of COVID-19 at the beginning of the pandemic, it provides insights that can help inform preventative measures for future possible pandemics and other major life-disrupting events. However, further work is necessary to fully comprehend how parents and couples have been impacted by COVID-19 and to better inform intervention programs to assist intimate partner relationships and families with the potential long-term repercussions of the pandemic. Results from the current study support the development of prevention strategies to reduce the use of alcohol as a coping mechanism for life-disrupting events. Public health initiatives may focus on enhancing family functioning to reduce IPV or increasing resource allocation to address maladaptive alcohol use and IPV. Moreover, preventative programming targeting adolescents may be beneficial, given the onset of IPV and alcohol use during this developmental period. Finally, implementing programming to support the adoption of more problem-focused coping strategies in the early stages of large-scale disruptions may be a worthwhile effort to reduce the risk of alcohol misuse.
